# Of the Causes, Nature, and Treatment of Palsy and Apoplexy: Of the Forms, Seats, Complications, and Morbid Relations of Paralytic and Apoplectic Diseases

**Published:** 1850-12

**Authors:** 


Art. V.—*Of the Causes, Nature, and Treatment of Palsy and Apoplexy: of the Forms, Seats, Complications, and Morbid Relations of Paralytic and Apoplectic Diseases.* By James Copland, M. D., F. R. S., &c. &c. Philadelphia: Lea & Blanchard. 1850.

Dr. Copland says: "A considerable part of the following *Treatise* was published many years ago in the first and third volumes of the author's Dictionary of Practical Medicine, and several of the chapters on the connection of Paralytic and Apoplectic seizures, with other disorders, formed the Cröonian Lectures, for 1846, and 1847, at the College of Physicians."

This admirable work is filled with sound and valuable matter, of the utmost importance to the physician.—There are few safer guides in the philosophy of medicine than Dr. Copland, when he is not mounted on his jaded hobby of contagion. His great characteristics as a writer are fulness of matter, and minuteness of detail, on all subjects that attract his attention. The work before us is much the most satisfactory one on palsy and apoplexy, and their connection with other disorders, that we have seen, and the student of its pages will arise from its perusal, not only considerably enlightened upon the special subjects of the book, but upon a multitude of points that have relation to a mass of medical matters of the highest interest to the physician. The developments of the constitution of the blood in various diseased actions of the human economy are of great value. Alas! for the days of solidism. The errors of humoralism, and its truths were alike buried under the imposing structure of solidism, and by revealing the manifold and extravagant errors of the humoral pathology, the solidists were able to destroy the truth, and obscure the light of that system. Solidism was embraced in all its length and breadth, on the principle, that, if humoralism was untrue, its converse must of necessity be true. But enlightened means of investigation have shown that humoralism, with all its blunders, had a great deal of truth in its doctines, and that its present revealments are of the utmost importance to the successful physician. If any one doubts the truth of this, even under the instructive lessons of Andral, we do not see how he is to resist the overwhelming evidence contained in the work before us, amply sustained and extended as the evidences are by Mr. Paget's microscopic investigations. If any one wishes to see truth triumphatly vindicated, medical philosophy purified, and science made sure and invulnerable, we pray him to give all the powers of his mind to the study of Dr. Copland's book. He will find its doctrines perpetual fountains of light, springing up in his path, guarding his footsteps from darkness, and his course from error.

The book opens with a consideration of "the intimate connection of Palsy snd Apoplexy." The following are the titles of the various sections of the work:

Section 1. "Of the less complicated forms of Palsy or Primary and Simple Palsy." This is divided into nine subjects.

Section 2. "The uncomplicated forms of Apoplexy,—or, Primary and Simple Apoplexy."

Section 3. "Of the Association of Palsy and of Apoplexy with each other."

Section 4. "Of several diseases often preceding, inducing, and complicating Apoplexy and Palsy." This section is one of the most luminous medical essays we have ever read.

Section 5. "The Diagnosis and Prognosis of Apoplexy and Palsy."

Section 6. "The remote causes, or contingent occasions, and the pathological states producing Palsy and Apoplexy."

Section 7. "Of the treatment of Palsy and Apoplexy."

The titles of these sections will convey some idea of the contents of the book, but he who may acquire a knowledge of those contents from the book itself, will never regret any amount of time he may have bestowed upon the study.

